# Short-Term Health Impact Assessment of Urban PM_10_ in Bejaia City (Algeria)

**DOI:** 10.1155/2016/8209485

**Published:** 2016-08-10

**Authors:** Fatima Benaissa, Cara Nichole Maesano, Rezak Alkama, Isabella Annesi-Maesano

**Affiliations:** ^1^Sorbonne Universités, UPMC Univ Paris 06, INSERM, Institut Pierre Louis d'Epidémiologie et de Santé Publique (IPLESP UMRS 1136), Epidemiology of Allergic and Respiratory Diseases (EPAR) Department, Medical School Saint-Antoine, 75012 Paris, France; ^2^Department of Environmental and Biological Sciences, Bejaia University, 06000 Bejaia, Algeria; ^3^Biological Department, Boumerdes University, 35000 Boumerdes, Algeria; ^4^Electrical Engineering Laboratory, Bejaia University, 06000 Bejaia, Algeria

## Abstract

We used Health Impact Assessment (HIA) to analyze the impact on a given population's health outcomes in terms of all-causes mortality and respiratory and cardiovascular hospitalizations attributable to short-term exposure to particulate matter less than 10 *μ*m diameter (PM_10_) in Bejaia city, for which health effects of air pollution have never been investigated. Two scenarios of PM_10_ reduction were considered: first, a scenario where the PM_10_ annual mean is decreased by 5 *µ*g/m^3^, and then a scenario where this PM_10_ mean is decreased to 20 *µ*g/m^3^ (World Health Organization annual air quality guideline (WHO-AQG)). Annual mean level of PM_10_ (81.7 *µ*g/m^3^) was calculated from objective measurements assessed* in situ*. Each year, about 4 and 55 deaths could be postponed with the first and the second scenarios successfully. Furthermore, decreasing PM_10_ annual mean by 5 *µ*g/m^3^ would avoid 5 and 3 respiratory and cardiac hospitalizations, respectively, and not exceeding the PM_10_ WHO-AQG (20 *µ*g/m^3^) would result in a potential gain of 36 and 23 per 100000 respiratory and cardiac hospitalizations, respectively. Lowering in current levels of PM_10_ has a nonnegligible impact in terms of public health that it is expected to be higher in the case of long-term effects.

## 1. Introduction

Short-term variations in air pollution have been associated with mortality from various causes in cities all over the world [1–10]. These associations include all-cause mortality [11–15], respiratory mortality [16–20], and cardiac mortality [21–23]. Among air pollutants, suspended particulate matter (PM) is extensively recognized as the most important air pollutant in terms of human health effects considering that many epidemiological studies substantiate significant associations between concentration of PM in the 2 air and adverse health impacts [4, 5, 24–28].

Health impacts assessment of Air Pollution (HIA-AP) is a method encouraged by WHO [29], whose aim is to provide the number of health events that could be prevented by reducing air pollutant levels in the target population.

Studies on health impact of air pollution carried out in Algeria are few and limited to Algiers where air pollution is monitored through one air quality (AQ) station. In other regions, like Bejaia city (~200000 inhabitants) there are no AQ measuring stations. Yet, the constantly increasing number of vehicles, their age (average of 8.5 years), and the tendency to dieselization (52.9% in 2014) are reasons that make Bejaia vehicle fleet a major source of air pollution. In a precedent paper [30], we have presented a descriptive study of the impact of air pollution as assessed through vehicles counts on Bejaia population. We have shown that at the population level exposure to vehicle air pollution is a cause of increased prevalence of respiratory diseases. No objective assessment of air pollutants levels was available at that time.

In this paper, we present for the first time results for HAI-AP in Bejaia city. Our HAI provided estimates of the number of health events attributable to PM_10_ in the target population. We proposed two scenarios: we first considered a scenario where the PM_10_ annual mean is decreased by 5 *μ*g/m^3^ and then a scenario where the PM_10_ annual mean is decreased to 20 *μ*g/m^3^ (WHO-AQ). To this extent, we conducted an* ad hoc* campaign to objectively assess PM_10_ levels.

## 2. Methods

The HIA-AP method as previously described with relative risks from the dose-response functions as those used in the Aphekom City Report for the town of Marseilles [33] for short-term mortality and morbidity. The adverse health outcomes considered in this analysis include mortality (all causes except external causes, adults) and morbidity (respiratory and cardiac hospitalizations). RR = relative risk/10 *µ*g/m^3^ (Table 1) taken from Aphekom City Report Marseilles.

Two scenarios were considered (Table 2): the annual mean level of PM_10_ is decreased by 5 *µ*g/m^3^, and PM_10_ annual mean level is decreased to the WHO annual air quality guideline (20 *µ*g/m^3^).

### 2.1. Study Area

To satisfy the assumption of homogeneity in air quality, only urban areas were retained. There is a minimum geographical area compatible with the extraction of mortality data and hospital admissions which is the town of Bejaia, located 15 m above the sea (36° 45′ 00′′ N and 5° 04′ 00′′ E).

### 2.2. PM_10_ Assessments

Due to lack of data on the quality of outdoor air in Bejaia, we chose to make by ourselves* ad hoc* outdoor pollution measurements. These measurements were performed for 4 months (04/08/2015 to 07/14/2015), this period coinciding with atmospheric stagnant conditions. In their study, Alkama et al. [34], studying seasonal variations of air pollution (CO, NO, and SO_2_), showed that measured concentrations are strongly correlated with industrial activity and heavy traffic in this city: maximum values were observed in the summer period, while minimum values were measured in winter, a period in which wind speed produced high atmospheric dilution.

The underlying assumption is that air pollution during the given period was representative of the usual air pollution.

**Figure 1:**
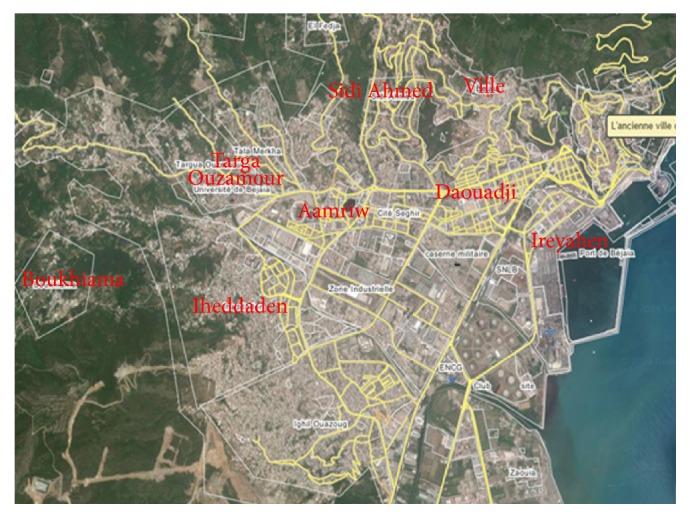
Bejaia: map of sampling sites.

PM_10_ assessments were conducted in 8 stations in Bejaia city using a portable AEROCET 531S Particle Mass Profiler and Counter.  Figure 1 presents the locations which were selected. PM measurements were carried out during the period. However, the analysis will focus on PM_10_ because of the availability of PM_10_ dose-response function in the literature. The AEROCET 531S is a full-featured, battery operated, handheld particle counter or mass monitor. The sample time is fixed at 60 seconds. The instrument store sample events. These records can be viewed on the display or transferred to a computer via USB or RS232. Data files were imported into an Excel® formatted output.

Before sampling, the AEROCET-531 monitor was tested with a zero filter and flow meters to ensure proper functioning of the monitor. During sampling, the instrument was placed at a height of 1.5 m from the soil and at a distance of 4 m from the road.

### 2.3. Health Data

Mortality data were obtained from both the Statistics Service of Directorate Planning and Regional Development (Direction de Planification et de l'Aménagement du Territoire: DPAT) and Directorate of Health and Population (DSP: Direstion de la Santé Publique) of Bejaia. Table 2 describes population indicators and mortality rates for the period of analysis.

Data on hospitalization for cardiovascular and respiratory diseases were provided by the Pneumophtisiology Department at the Frantz Fanon Hospital.

Table 3 summarized collected health data.

## 3. Results

### 3.1. Air Quality

**Figure 2:**
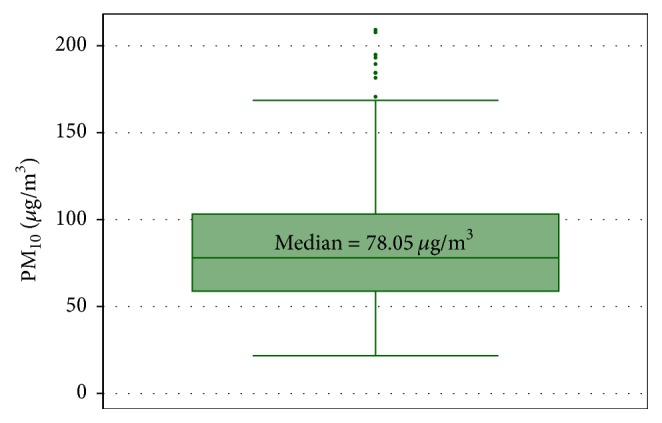
PM_10_ concentrations in *µ*g/m^3^.

Figure 2 shows the distributions for PM_10_. Most of the values fall around 81.7 *µ*g/m^3^, the mean of PM_10_ except for a few outliers.

### 3.2. Health Data

#### 3.2.1. Total Mortality

Among 1386 all-cause deaths in 2014 (Table 3), 4 deaths per year could be avoided according to the scenario of a reduction in annual PM_10_ levels of 5 *µ*g/m^3^ and 55 deaths according to the scenario of a reduction in annual PM_10_ levels to the WHO-AQG (20 *µ*g/m^3^) (Table 4).

#### 3.2.2. Morbidity (Hospital Admissions)

**Figure 3:**
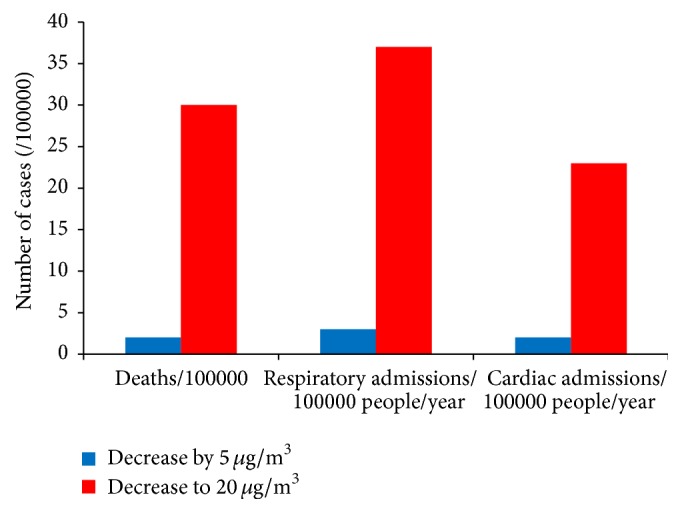
Potential benefits of reducing annual PM_10_ levels on mortality and on cardio respiratory morbidity in Bejaia city.

Decreasing PM_10_ annual mean by 5 *µ*g/m^3^ would avoid 5.2 and 3.2 respiratory and cardiac hospital admissions, respectively (Table 4). If the annual average of PM_10_ (87.8 *µ*g/m^3^) did not exceed the WHO-AQG (20 *µ*g/m^3^), there would be a decrease, or Δ*X*, of 67.8 *µ*g/m^3^. In this case the potential gain would be 36 per 100,000 and 23 per 100,000 for respiratory and cardiac hospital admissions, respectively (Figure 3).

## 4. Discussion

High levels of PM were observed in Bejaia, as over the sampling period the level average exceeded the WHO-AQG (20 *µ*g/m^3^) by 67.7 *μ*g /m^3^.

The specific Health Impact Assessment for Bejaia found that a significant health gain would be achieved by lowering annual means levels of PM_10_. In particular, compliance with WHO-AQG for PM_10_ (20 *µ*g/m^3^) would induce large benefits on mortality and cardiorespiratory hospital admissions. For the reduction scenario level of 5 *µ*g/m^3^, the number of avoidable deaths is 2 per 100,000 capita (0.3%) of the total mortality. This result is similar to that found in Marseilles. This town was taken in comparison because we used the same concentration-response function [13]. However for the second reduction scenario of compliance with WHO-AQG for PM_10_ (20 *µ*g/m^3^), the result of 29 avoided deaths per 100,000 far exceeds the number of deaths found in Marseilles (5 deaths per 100000).

At the same time, we estimated that a reduction of 5 *µ*g/m^3^ in the existing PM_10_ levels could diminish by about 3 per 100000 respiratory hospitalizations against 6 per 100000 in Marseilles and 36 per 100000 against 11 in the second scenario. Furthermore, according to the first scenario almost 2 (1.735) per 100000 cardiac hospitalizations could be avoided against 4 per 100000 in Marseilles. However, 23 per 100000 cardiac hospitalizations were avoided in the second scenario against 8 per 100000 in Marseilles.

From the public health point of view, our estimations clearly indicate that it is insufficient to reduce the PM_10_ concentrations by 5 *µ*g/m^3^ in Bejaia and that it has to be reduced to the WHO-AQG.

HIA with long-term effects will provide a better estimation of the strengths of reducing air pollution.

This is an approach by approximation. Our proxy is to take Marseille comparable to Bejaia, certainly more than any other European or American city with concentration-response function. However, we cannot discard the hypothesis of an underestimation of risk in our study in the absence of a local concentration-response function because the air quality in Bejaia is different from Marseilles.

Here we account only for PM_10_ and HIA-AQ can be used to estimate HI with other pollutants. Depending on the availability of the measuring tools, there has been only 4 months of measurement. We thus plan to take a minimum of one year' measurements. In future work we aim to include additional air quality data, for example, ozone and PAH, and focus more on fine PM and its components. We have to take more urbanistic typology and characteristics (not only car fleet characterization) for air pollution modelling in there.

This assessment on public health impacts of current patterns of PM_10_ in Bejaia city is the first one. Our investigations into this area are in progress to confirm our hypothesis. We claim to do further analyses on pollution and health impacts not only on a minimum geographical area of Bejaia city but also on others areas.

## 5. Conclusion

We observed for the first time that, in the urban zones of Bejaia, annual average PM_10_ levels exceeded the WHO limit of 20 *µ*g/m^3^, the EU limit of 40 *µ*g/m^3^, and the Algerian limits of 80 *µ*g/m^3^. These excess concentrations were responsible for 29 deaths, 36 respiratory hospitalizations, and 23 cardiac hospitalizations per 100,000 in 2014.

In conclusion, policies for the reduction of air pollution appear to be necessary, and their implementation will be rewarding in terms of the protection and improvement of individual and community health and installation of a monitoring and measuring site of air pollution is the basis of these policies.

## Figures and Tables

**Table 1:** Relative risks for nonexternal all-cause mortality and cardiorespiratory hospitalizations for PM_10_.

HIA	PM_10_ measures	Health outcome	RR/10 *µ*g/m^3^ ± [IC] 95%	*β*	References
Short-term	Annual mean	Nonexternal all-cause mortality	1.006 ± 0.002	0.000598207 ± 0.0001986	[31]
		Respiratory hospitalizations	1.0114 ± 0.0002	0.001133551 ± 0.000520	[32]
		Cardiac hospitalisations	1.006 ± 0.002	0.000598207 ± 0.0001986	[32]

*β* = ln⁡(RR)/10.

*Table taken from Aphekom City Report Marseilles*.

**Table 2:** Summary scenarios of PM_10_.

Scenarios	Health outcome indicator
(i) Decrease by 5 *µ*g/m^3^ in PM_10_ (ii) Decrease to 20 *µ*g/m^3^ in PM_10_	(i) Number of deaths avoided/year (ii) Number of respiratory admissions avoided/year (iii) Number of cardiac admissions avoided/year

GV_WHO_: Guide Value of World Health Organization.

**Table 3:** Health outcome and relative risk used in this HIA in 2014.

Health outcome data		Population data according to age	
Nonexternal all-cause mortality (all ages)	1386	All ages	185882
Respiratory hospitalizations (older than 65)	918	>65 years	16383
Cardiac hospitalizations (older than 65)	1080	[16–59]	125700

**Table 4:** Annual number of health outcomes.

Health outcome data	All-cause mortality		Respiratory hospital admissions		Cardiac hospital admission	
Scenarios	Δ*y* = deaths avoided/year [IC 95%]	Deaths/100000 inhabitants [IC 95%]	Δ*y* = admissions avoided/year [IC 95%]	Admissions/100000 people/year [IC 95%]	Δ*y* = admissions avoided/year [IC 95%]	Admissions/100000 people/year [IC 95%]
Decrease by 5 *µ*g/m^3^	4 ± 1	2 ± 1	5 ± 2	3 ± 1	3 ± 1	2 ± 1
Decrease to 20 *µ*g/m^3^	55 ± 18	30 ± 10	68 ± 30	37 ± 16	43 ± 21	23 ± 11

## References

[1] Dominici F. (2004). Time-series analysis of air pollution and mortality: a statistical review. *Synopsis of Research Report*.

[2] Wong C.-M., Vichit-Vadakan N., Kan H. (2008). Public Health and Air Pollution in Asia (PAPA): a multicity study of short-term effects of air pollution on mortality. *Environmental Health Perspectives*.

[3] Ballester F., Medina S., Boldo E. (2008). Reducing ambient levels of fine particulates could substantially improve health: a mortality impact assessment for 26 European cities. *Journal of Epidemiology & Community Health*.

[4] Orru H., Teinemaa E., Lai T. (2009). Health impact assessment of particulate pollution in Tallinn using fine spatial resolution and modeling techniques. *Environmental Health: A Global Access Science Source*.

[5] Wu S., Deng F., Niu J., Huang Q., Liu Y., Guo X. (2010). Association of heart rate variability in taxi drivers with marked changes in particulate air pollution in Beijing in 2008. *Environmental Health Perspectives*.

[6] Sousa S. I. V., Pires J. C. M., Martins E. M., Fortes J. D. N., Alvim-Ferraz M. C. M., Martins F. G. (2012). Short-term effects of air pollution on respiratory morbidity at Rio de Janeiro-part II: health assessment. *Environment International*.

[7] Bell M. L., Zanobetti A., Dominici F. (2013). Evidence on vulnerability and susceptibility to health risks associated with short-term exposure to particulate matter: a systematic review and meta-analysis. *American journal of epidemiology*.

[8] Kloog I., Ridgway B., Koutrakis P., Coull B. A., Schwartz J. D. (2013). Long- and short-term exposure to PM2.5 and mortality: using novel exposure models. *Epidemiology*.

[9] Hoek G., Brunekreef B., Goldbohm S., Fischer P., Van Den Brandt P. A. (2002). Association between mortality and indicators of traffic-related air pollution in the Netherlands: A Cohort Study. *The Lancet*.

[10] Shin H. H., Stieb D. M., Jessiman B. (2008). A temporal, multicity model to estimate the effects of short-term exposure to ambient air pollution on health. *Environmental Health Perspectives*.

[11] Gouveia N., Fletcher T. (2000). Time series analysis of air pollution and mortality: effects by cause, age and socioeconomic status. *Journal of Epidemiology and Community Health*.

[12] Middleton N., Yiallouros P., Kleanthous S. (2008). A 10-year time-series analysis of respiratory and cardiovascular morbidity in Nicosia, Cyprus: the effect of short-term changes in air pollution and dust storms. *Environmental Health: A Global Access Science Source*.

[13] Pascal L. (2009). Effets à court terme de la pollution atmosphérique sur la mortalité. *Revue des Maladies Respiratoires*.

[14] Moolgavkar S. H., Mcclellan R. O., Dewanji A., Turim J., Luebeck E. G., Edwards M. (2013). Time-series analyses of air pollution and mortality in the United States: a subsampling approach. *Environmental Health Perspectives*.

[15] Mills I. C., Atkinson R. W., Kang S., Walton H., Anderson H. R. (2015). Quantitative systematic review of the associations between short-term exposure to nitrogen dioxide and mortality and hospital admissions. *BMJ Open*.

[16] Dockery D. W., Pope C. A. (1994). Acute respiratory effects of particulate air pollution. *Annual Review of Public Health*.

[17] Sunyer J., Schwartz J., Tobías A., Macfarlane D., Garcia J., Antó J. M. (2000). Patients with chronic obstructive pulmonary disease are at increased risk of death associated with urban particle air pollution: a case-crossover analysis. *American Journal of Epidemiology*.

[18] Liang W.-M., Wei H.-Y., Kuo H.-W. (2009). Association between daily mortality from respiratory and cardiovascular diseases and air pollution in Taiwan. *Environmental Research*.

[19] Filleul L., Zaghnoun A., Declercq C. (2015). Relation à court terme entre la pollution atmosphérique urbaine et la mortality respiratoire: la place des études temporelles. Exemple de l’étude de 9 villes (SPSA-9). *Revue des Maladies Respiratoires*.

[20] Loftus C., Yost M., Sampson P. (2015). Regional PM_2.5_ and asthma morbidity in an agricultural community: a panel study. *Environmental Research*.

[21] Le Tertre A., Medina S., Samoli E. (2002). Short-term effects of particulate air pollution on cardiovascular diseases in eight European cities. *Journal of Epidemiology and Community Health*.

[22] Mills N. L., Donaldson K., Hadoke P. W. (2009). Adverse cardiovascular effects of air pollution. *Nature Clinical Practice Cardiovascular Medicine*.

[23] Shah A. S. V., Lee K. K., McAllister D. A. (2015). Short term exposure to air pollution and stroke: systematic review and meta-analysis. *The British Medical Journal*.

[24] Englert N. (2004). Fine particles and human health—a review of epidemiological studies. *Toxicology Letters*.

[25] Martuzzi M., Krzyzanowski M., Bertollini R. (2005). Health impact assessment of air pollution: providing further evidence for public health action. *European Respiratory Journal*.

[26] Laden F., Schwartz J., Speizer F. E., Dockery D. W. (2006). Reduction in fine particulate air pollution and mortality: extended follow-up of the Harvard Six Cities study. *American Journal of Respiratory and Critical Care Medicine*.

[27] Samoli E., Peng R., Ramsay T. (2008). Acute effects of ambient particulate matter on mortality in Europe and North America: results from the APHENA study. *Environmental Health Perspectives*.

[28] Boldo E., Linares C., Lumbreras J. (2011). Health impact assessment of a reduction in ambient PM_2.5_ levels in Spain. *Environment International*.

[29] Bart O. (2004). *Outdoor Air Pollution: Assessing the Environmental Burden of Disease at National and Local Levels*.

[30] Benaissa F., Alkama R., Annesi-Maesano I. (2014). Assessment of air pollution impacts on population health in Bejaia city, Northern Algeria. *Iranian Journal of Public Health*.

[31] Anderson H. R., Atkinson R. W., Peacock J. L., Marston L., Konstantinou K. (2004). Meta-analysis of timeseries studies and panel studies of Particulate Matter (PM) and Ozone (O_3_). *Report of a WHO Task Group*.

[32] Atkinson R. W., Anderson H. R., Medina S. (2005). Analysis of all-age respiratory hospital admissions and particulate air pollution within the Apheis programme. *APHEIS Air Pollution and Information System. Health Impact Assessment of Air Pollution and Communication Strategy. Third-Year Report*.

[33] Pascal M., Corso M., Chanel O. (2013). Assessing the public health impacts of urban air pollution in 25 European cities: results of the Aphekom project. *Science of the Total Environment*.

[34] Alkama R., Ait Idir F., Slimani Z. (2006). Estimation and measurement of the automobile pollution: application to Bejaia case. *Global NEST Journal*.

